# Value priorities and value conflicts in patients with mental disorders compared to a general population sample

**DOI:** 10.1038/s41598-022-07758-4

**Published:** 2022-03-10

**Authors:** Elisabeth A. Arens, Muriel Christoffel, Ulrich Stangier

**Affiliations:** Clinical Psychology and Psychotherapy, Department of Psychology, Goethe University Frankfurt, Varrentrappstr. 40-42, 60486 Frankfurt am Main, Germany

**Keywords:** Psychology, Human behaviour, Psychiatric disorders

## Abstract

Personal values are considered as guiding principles for humans’ attitudes and behavior, what makes them an essential component of mental health. Although these notions are widely recognized, investigations in clinical samples examining the link between values and mental health are lacking. We assessed n = 209 patients with affective disorders, neurotic disorders, reaction to severe stress, and adjustment disorders and personality disorders and compared them to a stratified random sample (n = 209) drawn from the European Social Survey. Personal values were assessed using the Portraits Value Questionnaire. Severity of psychopathology was assessed using the Beck Depression Inventory and the Brief Symptom Inventory. Clinical participants showed a higher preference for the values power, achievement and tradition/conformity and a lower preference for hedonism compared to controls. Patients exhibited more incompatible value patterns than controls. Across diagnostic groups, patients with neurotic disorders reported incompatible values most frequently. Value priorities and value conflicts may have the potential to contribute to a better understanding of current and future actions and experiences in patients with mental disorders.

## Introduction

Personal values are defined as broad, desirable, and trans-situational goals^1^. They guide humans’ attitudes and behavior^2^, what makes them an essential component of mental health^3^. Accordingly, values have been referenced in central psychological theories of mental health (e.g.^4,5^,) and are part of diverse psychotherapeutic approaches (e.g.^6^,). Given this, it is surprising that values and their link to mental distress have to date almost exclusively been tested in non-clinical populations. Rather, most studies have used samples from the general population (e.g.^3^,) or student samples (e.g.^7^,) to demonstrate the link between values and mental health. Furthermore, it has been neglected that not only what goals individuals prioritize, but also how those values relate to each other (i.e., whether they are compatible or incompatible) may independently correlate with mental health. Only few investigations (e.g.^8^,) have focused on intraindividual value conflicts as additional aspect in the link between personal values and mental health.

**Figure 1:**
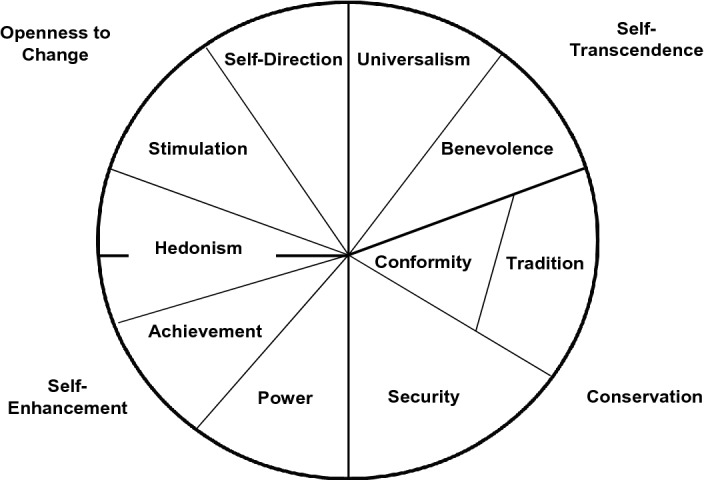
The value model of Schwartz (1992).

Schwartz^9^ proposed that the content and structure of personal values can be described by a metastructure of two dimensions: 1. self-transcendence vs. self-enhancement and 2. openness to change vs. conservation. Originally, Schwartz assumed ten motivationally distinct values that can be assigned to those dimensions: power, achievement, hedonism, stimulation, self-direction, universalism, benevolence, tradition, conformity, and security. However, several studies (e.g.^10^,) have provided evidence for a modified circular model of values, combining the factors conformity and tradition to one factor, due to their close relatedness. For this reason, in the present study the number of factors was reduced to 9 by forming a common factor from the conformity and tradition items (see Fig. 1).

Assumptions about the relationship between mental health and what personal values individuals hold, result in particular from the concept of deficit and growth values^1^. Deficit values (power, achievement, conformity, security, and tradition) are expected to have a negative relationship with mental health, as they indicate the lack of attainability of deficit-oriented goals such as order or security. In contrast, importance of growth-oriented values (universalism, benevolence, hedonism, stimulation, self-direction) increases when those goals are achieved.

In support of these assumptions a negative association with mental health was found particularly for the values of tradition, security and conformity, both in studies that explored subjective well-being^11^ and mental health symptoms^12,13^. In contrast, positive correlations with mental health were found for the values stimulation, hedonism and self-direction^13^. With regard to the values of achievement, power, benevolence and universalism, however, study results available to date are inconsistent. While in a German student sample achievement correlated positively with depression^12^, achievement and power correlated positively with mental health in a Russian sample^13^. For benevolence and universalism, a negative association with depression was found in a Russian and Chinese sample^13^, while in another study universalism and benevolence showed no association with mental health^7^.

Overall, the relationship between personal values and mental health has been analyzed largely on the basis of subjective well-being and has produced sometimes contradictory results. Moreover, the few studies that investigated value priorities with respect to psychopathological symptoms largely refer to the general population rather than to clinical samples^3,13^. Thus, to date it is unclear whether patients with mental disorders differ in their prioritization of values compared to individuals from the general population.

Regarding intrapersonal value conflicts, Schwartz’s Theory of Basic Human Values^14^ postulates that values can be arranged in a circular structure according to their compatibility. That is, two adjacent value types are motivationally similar, i.e., supposed to be positively correlated, whereas opposing value types are supposed to be negatively correlated (see Fig. 1). Given this, Schwartz’s model predicts which values will be motivationally compatible and which values will conflict with one another. For example, pursuing achievement values typically conflicts with pursuing benevolence values, as seeking success for the self tends to obstruct actions aimed at enhancing the welfare of others who need one's help.

According to Festinger's theory of dissonance^15^, people have a fundamental need for consistency in their cognitions, e.g., their personal values. In line with this, Grawe's incongruence theory^5^ posits that motivational incongruence may be followed by the development of psychopathological symptoms and may contribute to the maintenance of mental disorders. Inconsistent values may impede goal-directed action and may lead to approach-avoidance conflicts, which in turn may promote the development of psychopathological symptoms^16^.

Empirical evidence for the significance of inconsistency in patients with mental disorders comes from studies showing a relationship between levels of incongruence and mental distress. For example, it has been shown that depressed patients show a significantly higher degree of incongruence both between different values and between their values and the perceived realization of these values as compared to controls^8^. However, to our knowledge, a systematic evaluation of conflicting value constellations across Schwartz´s dimensions and their link to mental distress in a clinical sample compared to a general population sample has not yet been conducted. Further, it has not been investigated whether different mental disorders vary in the prevalence of intrapersonal value conflicts.

## The present study

In summary, the current study aims to test its hypotheses by drawing from Schwartz’s model of personal values to further understand the relationship between value priorities, conflicting value constellations and mental distress experienced by patients with diverse mental health disorders compared to individuals from a general population sample. Further, the study will aim to investigate whether value priorities are different across mental health disorders and whether conflicting value constellations are more prominent in certain diagnoses than others. We focused on the following questions: (1) How do patients with mental disorders differ in their value priorities from individuals of the general population? (2) Do patients more often show incompatible value constellations, compared to individuals from the general population? And 3. Do value priorities and value constellations differ across different mental disorders?

## Methods

### Participants

The clinical sample consisted of N = 209 adults (62% female, mean age 36.38 years, SD = 13.07) who met the ICD-10 criteria for: Affective Disorders (F30–F39) (n = 67, 56.72% female); neurotic disorders (F40, F41, F42, F45) (n = 66; 54.55% female); stress, and adjustment disorders (F43) (n = 44; 68.18% female); personality and behavioral disorders (F60–F69) (n = 22; 72.73% female); and other disorders not assigned to these four groups (F00–F29, F50–F59, F70—F99) (n = 10). The composition of each of the diagnostic groups with regard to specific diagnoses are shown in Table 1.

**Table 1:** Prevalence of specific diagnoses across disorder groups.

Disorder groups	*N*
**Affective disorders**	
Major depression, single episode	10
Major depression, recurrent	40
Persistent depressive disorder	12
**Neurotic disorders**	
Phobic anxiety disorders	38
Obsessive compulsive disorders	9
Somatoform disorders	7
Other anxiety disorders	12
**Reaction to severe stress, and adjustment disorders**	
Posttraumatic stress disorder	24
Adjustment disorders	20
**Personality and behavioral disorders**	
Personality disorders	18
Impulse control disorders	4
**Other disorders**	
Eating disorders	4
Sexual dysfunction disorders	2
Alcohol use disorders	2
Schizophrenia	2

Between 2017 and 2019 patients were recruited from the adult outpatient clinic of the Goethe University Frankfurt and were diagnosed by structured clinical interviews, carried out by the respective therapists. Based on their primary diagnosis, patients were divided into different diagnostic groups. 36% (n = 75) of the patients were comorbid, i.e., they exhibited at least one additional diagnosis of the other diagnostic groups.

As age, gender and educational level have been shown to be associated with value priorities^17–19^ and diagnostic groups were not parallelized in terms of those parameters, we included age, gender and educational level as control variables when comparing different diagnostic groups.

All participants received detailed information about the study and gave their written informed consent.

The sample of the general population was taken from the European Social Survey (ESS) for Germany from 2016, which consists of 2852 participants. A stratified random sample of N = 209 respondents was drawn from the 2852 respondents based on age and gender criteria. The final stratified sample contained 79 males (38%) and 130 females (62%), including individuals aged from 18 to 76 years. The average age was 36. 77 years (SD = 13.49). There was a significant difference between the clinical sample and the general population sample in educational level, χ^2^(3) = 7.63, *p* = 0.05, with patients having higher education on average. Educational level was controlled for in all analyses.

### Instruments

#### Potraits Value Questionnaire (PVQ)

Personal values were measured using the short German version of the Portrait Values Questionnaire (PVQ;^10^. The short PVQ includes 21 short verbal portraits of different individuals, each gender-matched with the respondent. Each portrait describes a person’s goals, aspirations, or wishes that point implicitly to the importance of a value. For example: “Thinking up new ideas and being creative is important to him. He likes to do things in his own original way” describes a person for whom the value self-direction is important. For each portrait, respondents answer: “How much like you is this person?” on a scale from 1 = very much like me to 6 = not like me at all. Each person's responses were centered around their respective mean value to eliminate individual differences in the use of the scale and to reflect the relative importance of values. Internal consistencies of the nine scales ranged from α = 0.60 (scale tradition/conformity) to α = 0.74 (scale hedonism). As all scales contain only two or three items respectively and the definitions of each value type are relatively broad, high internal consistencies were not expected.

#### Brief Symptom Inventory (BSI)

The BSI^20^ is a questionnaire designed to assess psychological and physical distress. Respondents are asked to rate their subjectively perceived symptom distress over the past seven days on a five-point scale ranging from 0 = not at all to 4 = very much. In total, the BSI consists of 53 items, which can be assigned to nine scales. From these, three indices of global distress can be calculated, the Global Severity Index (GSI), the Positive Symptom Total (PST), and the Positive Symptom Distress Index (PSDI). In the present clinical sample, the internal consistencies were in an acceptable to good range, ranging from α = 0.66 (psychoticism) to α = 0.84 (depressiveness).

#### Beck Depression Inventory II (BDI II)

The German version of the Beck Depression Inventory^21^ was used to assess the severity of depressive symptoms in the clinical sample. The BDI II contains 21 questions about how the patient felt during the past week. Respondents are asked to select the most applicable from four response options. The answer choices are ranked by intensity. A summed score is calculated from the responses, which can range from 0 to 63. A score of 18 or greater is considered clinically significant. For the total scale, Cronbach's alpha was 0.91.

### Procedure

Approval from the ethics committee of the Goethe University Frankfurt am Main was obtained, that the present study did not have to undergo any further ethical review. All participants gave written informed consent. During the study period, all instruments were part of the standard diagnostic assessment of the outpatient clinic of the Goethe University Frankfurt that is performed at the start of each treatment. Questionnaires were completed on the computer without therapist supervision. Diagnoses of mental disorders were made on the basis of clinical interviews (SCID-I and SCID-II) conducted by the respective therapist. In the European Social Survey the PVQ was conducted in a face-to-face interview.

### Statistical analyses

A multivariate analysis of variance (MANOVA) was used to compare the clinical variables among the five clinical groups. In order to compare value preferences between patients and controls, a multivariate analysis of covariance (MANCOVA) was conducted with the nine centered value types as dependent variable, group as independent variable and age, gender and education as covariates. The variables that remained significant in the multivariate models were then used in a discriminant analysis to test their discriminative power with regard to group classification. To contrast value conflicts between groups, chi-square tests of independence for each of the two value dimensions were performed to examine the relation between group (patients and controls; patients of different diagnostic groups) and compatible vs. incompatible value patterns.

### Ethics approval and consent to participate

All procedures followed were in accordance with the ethical standards of the responsible committee on human experimentation [institutional and national] and with the Helsinki Declaration of 1975, as revised in 2000. Approval from the ethics committee of the Goethe University Frankfurt am Main was obtained. All study participants gave written informed consent.

### Consent for publication

Neither the article nor portions of it have been previously published elsewhere. The manuscript is not under consideration for publication in another journal. All authors consent to the publication of the manuscript.

## Results

### Clinical characteristics of mental health patients

First, we analyzed clinical characteristics of mental disorder patients, who had filled out the BSI and BDI-II. Results indicated that there was a high general psychopathological symptom load across mental disorder groups, with an average Global Severity Index (GSI) of *M* = 0.93 (*SD* = 0.5). An average BDI Score of *M* = 20.46 (*SD* = 11.1) pointed to a moderate level of depressive symptoms across groups. Table 2 shows the results of the clinical measurements for each of the different groups. Compared to the other groups, patients with personality disorders exhibited significantly higher scores in almost all clinical scales. Patients with affective disorders exhibited higher levels of depression symptoms in both clinical measures (BDI, BSI), compared to patients with neurotic disorders and patients with reaction to severe stress, and adjustment disorders.

**Table 2:** Clinical measures in groups of different mental disorders.

Clinical scales and indices	Mental disorder group					Scheffé post hoc comparisons
	1.Affective disorders (n = 67)	2.Neurotic disorders (n = 66)	3. Reaction to severe stress, and adjustment disorders (n = 44)	4.Personality disorders (n = 22)	5.Other disorders^a^ (n = 10)	
BDI-II, sumscore (SD)	22.5 (10.1)	16.8 (10.5)	16.1 (8.8)	27.1 (11.9)	16.3 (14.5)	1 > 2, 3 p* 4 > 2,3 p***
**BSI scales, mean (SD)**						
Depression	1.2 (0.8)	0.9 (0.7)	0.7 (0.6)	1.7 (0.9)	1.1 (1.2)	1 > 3p* 4 > 2,3 p***
Somatization	0.7 (0.1)	0.8 (0.1)	0.7 (0.1)	0.8 (0.1)	0.6 (0.7)	ns
Phobic Anxiety	0.4 (0.5)	0.7 (0.7)	0.6 (0.9)	0.80 (0.77)	0.58 (0.73)	ns
Interpersonal Sensitivity	1.28 (0.11)	1.15 (0.11)	0.8 (0.1)	2.1 (0.2)	1.1 (1.1)	4 > 1p**,2,3p***
Obsessive–Compulsive	1.3 (0.1)	1.1 (0.1)	1.1 (0.1)	1.7 (0.1)	1.1 (1.1)	4 > 2,3p*
Psychoticism	0.7 (0.6)	0.5 (0.5)	0.5 (0.5)	1.1 (0.9)	0.8 (0.9)	4 > 2,3p**
Paranoid Ideation	0.8 (0.1)	0.6 (0.1)	0.6 (0.1)	1.2 (0.1)	1.1 (1.1)	4 > 2p**, 3p*
Hostility	0.8 (0.1)	0.6 (0.1)	0.7 (0.1)	1.5 (0.1)	0.7 (0.9)	4 > 1,2,3p***
Anxiety	0.9 (0.1)	0.9 0.1)	0.9 (0.1)	1.2 (0.1)	0.7 (0.6)	Ns
GSI	0.9 (0.5)	0.8 (0.5)	0.7 (0.5)	1.3 (0.6)	0.8 (0.7)	4 > 2,3p**
PSDI	1.7 (0.6)	1.6 (0.5)	1.6 (0.5)	2.1 (0.5)	1.7 (0.6)	4 > 1 p*, 2,3p**
PST	27.5 (1.2)	25.2 (1.3)	23.1 (1.6)	31.2 (2.2)	22.9 (12.9)	4 > 3 p*

BDI-II, Beck Depression Inventory II; BSI, Brief Symptom Inventory; GSI, Global Severity Index; PSDI, Positive Symptom Distress Index; PST, Positive Symptom Total.

*p ≤ .05; p** ≤ .01; p*** ≤ .001.

^a^Group “other disorders” was not included in statistical analyses due to small sample size.

### Value priorities in the clinical vs. general population sample

In a first step, a MANCOVA was conducted with the nine centered value types as dependent variable, group as independent variable and age, gender and education as covariates. It was analyzed whether patients and individuals from the general population differed significantly in their value priorities. Analyses revealed that after controlling for age, gender and education, group still had an significant effect on values priorities, explaining 13% of the variance, *F*(8, 476) = 8.03, *p* < 0.001, η^2^ = 0.13. The covariates age *F*(9, 408) = 7.23, gender *F*(9, 408) = 3.78, and educational level *F*(9, 408) = 3.52 also reached significance, all *p*s < 0.001. Post-hoc comparisons revealed that the patient sample differed significantly from the general population showing a stronger preference for the values power, achievement and tradition/conformity and a weaker preference for hedonism. According to Cohen's^22^ conventions, these are small to medium effects (see Table 3).

**Table 3:** Results of MANCOVA model.

Personal values	Clinical sample Mean^a^ (SD)	General population sample Mean^a^ (SD)	Group effect		Goodness of fit indices	
			*F* statistic	*P* value	Adjusted *R*^2^	Partial n^2^ (for group factor)
Power	− 0.7 (0.9)	− 1.2 (0.8)	35.55	< .001	0.08	0.07
Achievement	− 0.1 (1.1)	− 0.5 (0.9)	23.08	< .001	0.06	0.04
Hedonism	− 0.2 (0.9)	0.1 (0.9)	25.37	< .001	0.10	0.05
Stimulation	− 0.6 (0.9)	− 0.6 (0.9)	0.12	.723	0.02	0.00
Self-direction	0.5 (0.8)	0.6 (0.7)	0.05	.824	0.01	0.00
Universalism	0.6 (0.7)	0.7 (0.6)	1.54	.215	0.02	0.00
Benevolence	1.1 (0.7)	1.1 (0.5)	0.48	.489	0.01	0.00
Security	0.120 (0.9)	0.1 (0.8)	0.69	.405	0.01	0.00
Tradition/conformity	− 0.5 (0.9)	− 0.3 (0.9)	12.95	< .001	0.02	0.02

^a^Mean values are centered.

A discriminant analysis was conducted with the variables that were outstanding in the MANCOVA, i.e., the values power, achievement, hedonism and tradition/conformity were entered as predictor variables and group as dependent variable. Box’s M indicated that criteria for equality of covariance matrices were met. With a Wilks lambda of 0.855, the four value types discriminated significantly between patients and the general population, χ^2^(4) = 77.35, *p* = 0.001, accounting for 15% of between group variability. The closer analysis of the structure matrix revealed all four predictors as significant, all *p*s < 0.001. The classification matrix showed that overall 70.2% of the cases were correctly classified. Discriminant function coefficients and classification results are shown in Table 4.

**Table 4:** Canonical discriminant function coefficients and classification results.

	Canonical discriminant function coefficients		Classification results^a^		
	Function		Predicted Group membership		
	1	Clinical vs. General	Clinical	General	Total
Power	− .494				
Achievement	− .322				
Hedonism	.635	Clinical %	70.6	29.4	100
Tradition/Conformity	.469	General Population	30.2	29.8	100

^a^70.2% of original grouped cases correctly classified. A total of 418 cases were entered.

In a last step we also investigated whether patients with and without comorbid diagnoses differed with respect to their value preferences. Groups did not differ, *F*(9, 199) = 0.81, *p* = 0.613, η^2^ = 0.03.

### Compatible versus incompatible value patterns in the clinical vs. general population sample

In order to contrast compatible vs. incompatible value patterns, we calculated an incompatibility score based on the number of agreements with conflicting values, i.e., simultaneous agreement with values assigned to the two opposite poles of the dimension, respectively. The classification into the categories *compatible* vs. *incompatible* was based on the following criteria: On the dimension openness to change vs. confirmation, participants’ value pattern was classified as incompatible when they agreed with at least two values of the pole openness (i.e., self-direction, stimulation, hedonism) and with at least two values of the pole conservation (i.e., tradition/conformity, security). Although hedonism shares elements of both openness to change and self-enhancement, for the current analysis hedonism was assigned to the pole openness to change, as hedonism and tradition have frequently been outlined as conflicting values^14,23^. On the dimension self-transcendence vs. self-enhancement, participants’ value pattern was classified as incompatible when they agreed with at least one value of the pole self-enhancement (i.e., achievement, power) and with at least one value of the pole self-transcendence (i.e., benevolence, universalism). On both dimensions, agreement was defined by a rating score exceeding a cutoff score defined by the 75th percentile of the respective scale.

A chi-square test of independence for each of the two dimensions was performed to examine the relation between group and compatible vs. incompatible value pattern. On both, the openness to change vs. confirmation dimension, χ^2^ (1, *N* = 418) = 3.67, *p* = 0.049, and self-transcendence vs. self-enhancement dimension, χ^2^ (1, *N* = 418) = 11.97, *p* = 0.001, individuals from the clinical sample were significantly more likely to have incompatible value patterns than individuals from the general population.

### Value priorities and value incompatibility across different disorder groups

A MANCOVA was calculated with value types as dependent variables, disorder group as independent factor and age, gender and education as covariates. Due to its small sample size, the group “other disorders” was not included. No significant effect was found for group of disorders, *F*(9, 189) = 0.90, *p* = 0.613, η^2^ = 0.04, indicating that diagnostic groups did not differ in their value priorities.

**Figure 2:**
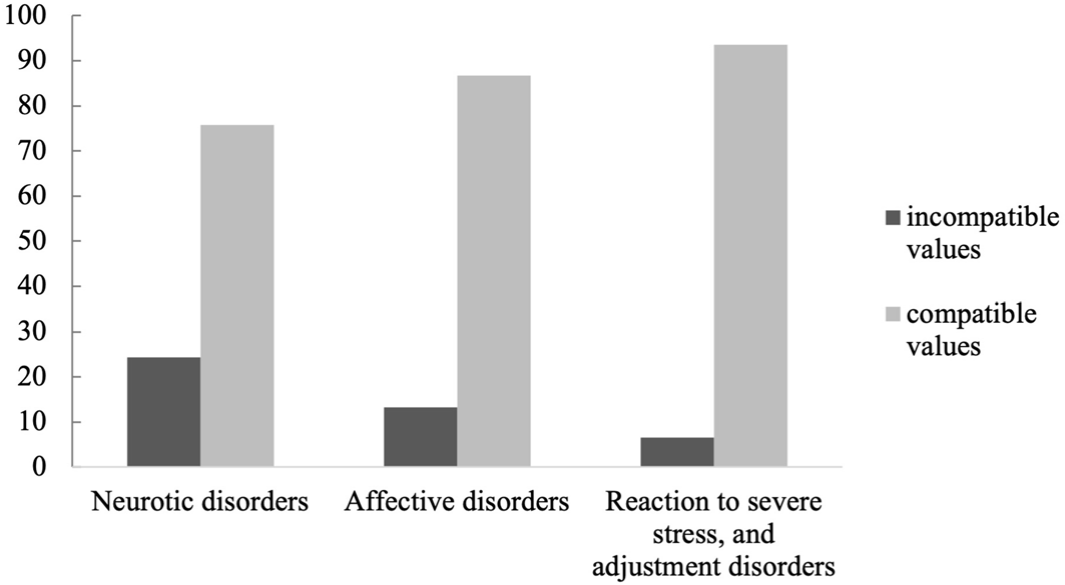
Percentages of individuals with neurotic (n = 66), affective (n = 67) and reaction to severe stress, and adjustment disorders (n = 44) having compatible vs. incompatible values on the dimension self-transcendence versus self-enhancement.

A chi-square test was performed to examine the relation between disorder group and compatible vs. incompatible value patterns. We additionally excluded the group “personality disorders” due to its small sample size. On the self-transcendence vs. self-enhancement dimension analyses revealed a significant effect, χ^2^ (2, *N* = 177) = 7.14, *p* = 0.028. Post hoc tests showed that individuals with neurotic disorders were significantly more likely to have an incompatible value pattern on that dimension than individuals with stress, and adjustment disorders χ^2^ (1, *N* = 110) = 6.13, *p* = 0.013 (see Fig. 2). Depressive and stress, and adjustment disorders did not differ in their frequencies of incompatible value patterns, χ^2^ (1, *N* = 111) = 1.38, *p* = 0.240. There was also no significant difference between depressive and neurotic disorders, albeit there was a trend towards significance, χ^2^ (1, *N* = 133) = 2.86, *p* = 0.090. On the openness to change vs. confirmation dimension analyses revealed no significant effect, χ^2^ (2, *N* = 177) = 0.98, *p* = 0.611.

## Discussion

The aim of the current study was to investigate whether patients with mental disorders (1) hold different values compared to individuals from the general population (2) have more intrapersonal value conflicts than the general population (3) differ from each other, depending on their diagnosis, in value priorities and intrapersonal value conflicts.

### Differences in values between patients and controls

Analyses revealed significant differences between mental health patients and the reference group. More specifically, differences were found between the groups in the values power, achievement, tradition/conformity and hedonism. Post-hoc analysis revealed that individuals with mental health disorders rated the importance of power, achievement and tradition/conformity higher and the importance of hedonism lower than the general population sample.

The results regarding power and tradition/conformity support previous findings showing a negative association between prioritizing those values and well-being^12^. Schwartz & Sortheix^1^ suggest that holding deficiency values like power and tradition/conformity expresses self-protective and anxiety control orientations. It is argued that individuals who feel unsafe and threatened emphasize those values, as their realization promises greater certainty. Uncertainty or intolerance of uncertainty is proposed to be a transdiagnostic causal mechanism of psychological difficulties^24^ and has been identified in several mental disorders^25,26^. Thus, prioritizing control and dominance over people and resources (power) as well as emphasizing social and cultural norms (tradition/conformity) might represent the attempt to increase one's own sense of security and controllability in patients across diagnostic groups.

Findings regarding achievement are consistent with other studies showing relations between achievement and psychopathological symptoms^12^. However, overall findings for achievement are inconsistent, with studies also showing positive relations to mental well-being^27,28^. Achievement values can be both, self-expansive (expressing competence) and self-protective (meeting social standards). In the context of mental disorders, a high prioritization of achievement might express self-protective motives, leading to dysfunctional behaviour. For instance, depression was related to dysfunctional types of perfectionism^29^ and inadequate high goal setting^30^. It has been argued that depressive affect might especially arise when individuals judge that they lack the efficacy to fulfill difficult goals but continue to strive for them for any sense of satisfaction or repair of self- worth^31^. Thus, in individuals with mental disorders the value achievement might be used for self-protection and repair of self-worth, what in turn may contribute to maladaptive behavior and mental distress symptoms.

In sum, current findings show that patients with mental disorders prioritize deficiency values more frequently than individuals from the general population. One clinical implication from these findings is that psychotherapists may explicitly focus on modifying feelings of helplessness and uncertainty and strengthen the patient’s sense of security, which may in turn allow a stronger orientation towards growth values.

### Value conflicts in patients and controls

Importantly, individuals with mental disorders did not only differ in the *type* of values they prioritized, but also in more frequent incompatible value patterns. It has been argued that inconsistent values may interfere with effective actions and might result in approach-avoidance conflicts^16^, associated with subsequent triggering of psychopathological symptoms. For instance, being caught in a conflict between emphasizing independence, and readiness for change on the one side, and emphasizing order, self-restriction, and resistance to change on the other side may have a debilitating effect on decision making and behavior. There is evidence demonstrating a link between sense of coherence and well-being within various clinical groups^32^. Our results indicate that conflicting value patterns, as they inhibit value-congruent behavior, may be crucial factors threatening psychological well-being in individuals with mental disorders. It has been noted that internal conflicts impede change in psychotherapy as changes might at least partially be experienced as threatening. Accordingly, some psychotherapeutic approaches have considered the resolution of internal conflicts as being central to the process of psychotherapy, such as experiential^33^, cognitive^34^, and motivational^5^ approaches. Thus, psychotherapy should not only address values as motivators for future behavior (e.g., as a directional component in behavioral activation) as it is the case in current approaches, e.g., Acceptance and Commitment Therapy^6^. Rather, they should also focus on the patient’s value constellations as potential indicators for conflicting needs that have to be resolved, e.g., via cognitive restructuring.

Our analyses have revealed that, across diverse diagnostic groups, patients showed a preference for power, achievement, as well as tradition/conformity. As those deficiency values are assumed to express self-protective and anxiety control orientations, these findings provide further evidence that uncertainty, or intolerance of uncertainty may be a transdiagnostic causal mechanism associated with a broad range of mental disorders (for a review see^23^). Further, they support the relevance of addressing personal values in psychotherapy—regardless of what diagnosis the patient suffers from.

### Comparison of value conflicts across diagnostic groups

In contrast to general value preferences, disorder specific effects were found for incompatible value constellations. On the self-transcendence vs. self-enhancement dimension individuals with neurotic disorders exhibited most frequently incompatible value patterns. That is, one the one hand those patients prioritized anxiety-avoidance values, i.e., reaching personal success according to social standards as well as gaining control over people and resources. On the other hand, they prioritized anxiety-free values, i.e., the preservation and enhancement of the welfare of other people and the environment. This ambivalence might mirror the conflict between approach-related drives (e.g., to seek positive social interactions) and avoidance-related drives (e.g., to prevent being humiliated) which is assumed to underly the dysfunction of neurotic disorders, e.g., anxiety disorders^35^. The current findings may indicate that such motivational conflicts are not only evident on the behavioral level but are also anchored on the superordinate level of personal values. When treating patients with neurotic disorders, psychotherapists may be especially alert to such cognitive conflicts.

## Limitations

There are several limitations of the present study that have to be mentioned: First, analyses were based on cross-sectional data, thus no causality can be derived for the relationship between value preferences, value conflicts and mental disorder symptoms. Sagiv and Schwartz^27^ discussed three possible mechanisms of how values and mental health might be related. First, values might contribute to individuals having certain attitudes and behaviors, which in turn are more favorable/unfavorable for mental health. Second, healthy values could directly contribute to the satisfaction of certain intrinsic motives, whereas unhealthy values may frustrate such important needs, which in turn could lead to dysfunctional compensatory activity. Whereas these first two mechanisms assume that pursuing particular values causally influences mental health a third mechanism hypothesizes the reverse causal direction, i.e., the level of mental health influences value priorities. For example, people who are satisfied with their lives are more likely to have emotional and cognitive resources to live their own lives in a self-determined way and to develop benevolence toward others. In contrast, people whose mental state is in less favorable conditions may lack the resources to pursue those values. They may focus on values instead the realization of which raises the prospect of security and relief from stress, e.g., conformity, tradition, power. Future research is needed, systematically investigating those potential mechanisms to further understand how personal values and mental health are related. Second, the current study was conducted in a clinical sample, of which over one third of the patients were comorbid. Clinical samples without comorbidity would have allowed to more carefully tease apart group differences directly attributable to certain diagnosis. However, we tested the impact of comorbidity by comparing patients with and without comorbid diagnoses and found no effect. Third, the clinical sample had a higher education on average than the general population sample. We therefore controlled in all analyses for education. A higher educational level in the clinical sample compared to controls is an unusual finding that could call into question the representativeness of the clinical sample. However, a closer look at the data showed that the educational level of the clinical sample corresponds to the typical educational distribution in Germany, whereas the ESS sample had an educational level somewhat below the average of the German population^36^. Reasons for this bias in the ESS sample need to be explored in a separate study, which is beyond the scope of this paper. Fourth, all of our measures relied on self-report, so that we cannot rule out the possibility that data may have been affected by social desirability bias. Future research should apply a more sophisticated study design, in which participants are unaware that their personal values are measured. Fourth, mental disorder symptoms were not measured in the general population, as the data were taken from the European Social Survey. Therefore, it cannot be ruled out that mental disorders were also present in this sample. This may have limited the between-group variance, underestimating the effects.

In conclusion, the present data point to an interesting link between value priorities, value conflicts and mental health. Mental disorders were shown to be characterized by a stronger preference for deficiency-oriented values and more incompatible value constellations. In a broader framework, personal values and value constellations should be integrated in a motivational theory contributing to a better understanding of current and future actions and experiences in patients with mental disorders.

## Data Availability

The datasets generated during and/or analysed during the current study are available from the corresponding author on reasonable request.
